# Soybean molasses: a promising substrate for the production of microbial oil by *Phaffia rhodozyma*

**DOI:** 10.1007/s00449-026-03312-y

**Published:** 2026-04-15

**Authors:** Shirley Denisse Ccori Ponce, Luiz Henrique Han, Luiz Antonio de Almeida Pinto, Carlos André Veiga Burkert, Janaina Fernandes de Medeiros Burkert

**Affiliations:** 1 Sensory Analysis and Quality Control Laboratory, School of Chemistry and Food, Federal University of Rio Grande, Rio Grande, 96203-900 Brazil; 2 Drying Laboratory and Industrial Technology Laboratory, School of Chemistry and Food, Federal University of Rio Grande, Rio Grande, 96203- 900 Brazil; 3 Bioprocess Engineering Laboratory, School of Chemistry and Food, Federal University of Rio Grande, Rio Grande, 96203-900 Brazil

**Keywords:** Unsaturated fatty acids, Oleaginous yeasts, Agro-industrial substrates, Bioactive pigments, Carotenogenic yeasts

## Abstract

Soybean molasses represents a promising substrate for the production of high-value bioproducts, particularly in the cultivation of *Phaffia rhodozyma*, a yeast classified as GRAS (Generally Recognized as Safe). Although this yeast is well known for carotenoid synthesis, its potential for lipid accumulation remains largely unexplored. In this study, oil production by *P. rhodozyma* was evaluated using soybean molasses as the sole carbon source. The yeast was cultured in a 5 L bench-scale bioreactor (100 g·L⁻¹ substrate, 300 rpm, 1 vvm, 25°C) and the biomass was subsequently spouted bed drying. Lipids were extracted following the Bligh and Dyer method. After 144 h, 12.59 g.L⁻¹ of biomass was obtained, containing 154.23 µg·g⁻¹ of astaxanthin and 85.91 µg·g⁻¹ of β-carotene. Lipid yield reached 34.9% (w.w^− 1^), corresponding to a C/N ratio of 44.39 in the cultivation medium. Fatty acid analysis revealed a predominance of oleic acid (66.98%), with a saturated/unsaturated ratio of 0.24, iodine value of 74 g.I_2_.100 g⁻¹, saponification value of 161 mg KOH.g⁻¹, acidity of 5.02 mg KOH.g⁻¹, and peroxide value of 3.19 mEq O₂.kg⁻¹. These results demonstrate the potential of *P. rhodozyma* cultivated on soybean molasses to produce microbial oil with high nutritional and functional value, favorable lipid composition, and oxidative stability, highlighting the need for optimization strategies in the oil recovery step to enable future applications in the food industry.

**Graphical abstract:**

**Figure:**
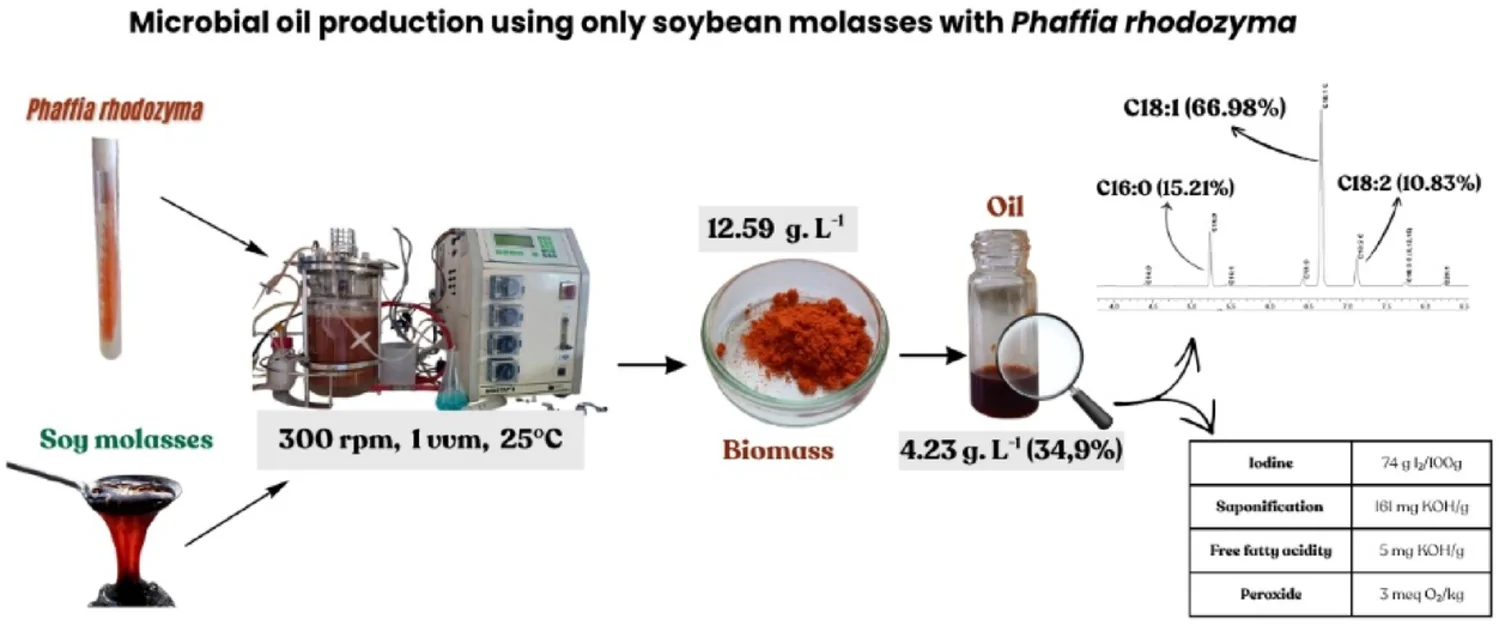

**Supplementary Information:**

The online version contains supplementary material available at https://doi.org/10.1007/s00449-026-03312-y.

## Introduction

The increase in the global population has intensified the demand for food, resulting in the depletion of natural resources, waste generation, and the expansion of agricultural practices to meet society’s needs. Among the most demanded products are oils, which are essential for both the food industry and biodiesel production, thereby intensifying competition for areas dedicated to the cultivation of oilseed crops [1].

The replacement of trans fats with healthier oils, promoted by World Health Organization (WHO) campaigns under the initiative “REPLACE” and reinforced by regulations in several countries, has increased the demand for oils rich in unsaturated fatty acids, which exhibit nutritional and technological effects beneficial to health, such as reducing low-density lipoprotein (LDL) and anti-inflammatory activity [2, 3]. Currently, the production of unsaturated oils still depends mainly on plant-based sources [4], facing challenges related to quality, diversification, and environmental impacts arising from monoculture practices [5, 6].

Biotechnological processes involving oleaginous yeasts have emerged as promising alternatives. About 160 oil-producing yeasts (≥ 20% lipids) have been described, and among them, red yeasts capable of producing carotenoids, such as *Phaffia rhodozyma*, stand out due to their antioxidant potential, attractive coloration, and GRAS (Generally Recognized as Safe) status, being mainly studied for the production of astaxanthin and β-carotene [7, 8]. Although the lipid potential of *P. rhodozyma* remains little explored, studies indicate that its lipids contain unsaturated fatty acids of high commercial value, combined with carotenoid content, giving it great potential for future applications [9].

Low-cost agro-industrial culture media have been increasingly investigated for the cultivation of *P. rhodozyma*. Among these, soybean molasses, a by-product of soybean protein processing, emerges as a promising alternative due to its high carbon content [10]. Furthermore, it stands out for its low cost (around USD 4.23 kg⁻¹) and high availability in Brazil, one of the world’s largest soybean producers [11, 12]. In soybean meal production, approximately 232 kg of molasses are obtained per ton, containing about 70% protein, as well as sugars, lipids, minerals, phenolic acids, and other phytochemicals [13]. Studies indicate that soybean molasses favors the growth of diazotrophic bacteria [10, 14, 15] and, most importantly, enhances biomass and carotenoid production by *P. rhodozyma*, potentially reducing culture medium costs by up to 90% compared to conventional media [11].

Although it has been previously demonstrated that the use of soybean molasses enhances biomass and carotenoid production, the effect of this substrate on lipid accumulation by *P. rhodozyma* cultivated with soybean molasses as the sole carbon source has not yet been investigated. To date, studies employing agroindustrial substrates have primarily focused on microorganisms widely recognized for their lipid-accumulating capacity or have emphasized carotenogenic potential.

Therefore, this study aimed to evaluate oil production by *Phaffia rhodozyma* cultivated in soybean molasses as the sole substrate in a bench-scale bioreactor, with partial characterization of the oil produced to assess both the sustainable potential of the substrate and the nutritional properties of the lipid.

## Materials and methods

### Microorganism, pre-inoculum, inoculum preparation, and culture conditions

The yeast *Phaffia rhodozyma*, strain NRRL Y-17,268, originating from the Northern Regional Research Laboratory (Peoria, IL, USA) and recognized as GRAS (Generally Recognized as Safe), was maintained on slanted agar containing agar (20 g.L⁻¹) in YM medium (glucose 10 g.L⁻¹, peptone 5 g.L⁻¹, yeast extract 3 g.L⁻¹, and malt extract 3 g.L⁻¹), and stored at 4 °C [16]. The cell resuspension (pre-inoculum) was prepared with 1 mL of 0.1% (v/v) peptone water, obtained by scraping the cells from the agar, and transferred to 9 mL of YM broth, being incubated at 25°C for 48 h. Then, the inoculum was prepared with 90 mL of YM medium and 10 mL of the pre-inoculum, incubated at 150 rpm, 25 °C, initial pH 6, until reaching a minimum cell density of 1×10⁸ cells.mL⁻¹, determined in a Neubauer chamber [17].

Batch cultivation was carried out in 5 L bench-scale bioreactor, with a working volume of 4 L, adding 3.6 L of agro-industrial medium containing soybean molasses (100 g.L⁻¹) as the sole substrate [11] and 10% inoculum, starting the cultivation with approximately 1×10⁷ cells.mL⁻¹, under the following conditions: 25 °C, initial pH adjusted to 6, 300 rpm, and 1 vvm [8].

### Characterization of the agro-industrial culture medium

Soybean molasses, used as the sole substrate in cultivation, was supplied by CJ Selecta, located in Araguari, Brazil, and kept refrigerated at 8 °C. The physical-chemical characterization followed the methodology of Oliveira; Michelon; Burkert [15]. which includes protein content determined by the Kjeldahl method, method 955.04 C of AOAC [18]; lipids by Soxhlet extraction [19], ash by muffle furnace incineration method 925.10 of AOAC [18], and carbohydrates by difference. The elemental analysis of carbon, hydrogen, and nitrogen (CHN) necessary to establish mass balances and evaluate the efficiency of nutrient conversion into biomass and metabolites was conducted in an elemental analyzer (Perkins Elmer 2400 Series II, USA), by combustion in pure oxygen, with detection and quantification of gases by thermal conductivity (TCD) [14].

### Determination of the volumetric oxygen transfer coefficient in the liquid phase (K_L_a) in the production medium

To determine the volumetric oxygen transfer coefficient (k_L_a) in the liquid phase, dissolved oxygen was initially removed from the uninoculated culture medium by sparging nitrogen into the bioreactor. Subsequently, aeration at 1 vvm and agitation at 300 rpm were applied, and the dissolved oxygen concentration was monitored in real time using an electrode, allowing k_L_a to be calculated based on the medium’s reoxygenation rate [14] The k_L_a coefficient was calculated using Eq. (1) below.

1 $${\text{ - k}}_{{\mathrm{L}}} {\mathrm{a}}{\mathrm{.t}}\,{\text{ = }}\,{\mathrm{ln}}\,{\mathrm{(}}\frac{{{\mathrm{C}}^{{\mathrm{*}}} {\text{ - DO}}}}{{{\mathrm{C}}^{{\mathrm{*}}} }}{\mathrm{)}}$$

Where: t= time (h), C*= concentration of oxygen at air–liquid equilibrium (corresponding to 100% DO), DO = concentration of dissolved oxygen in the medium (%).

### Determination of biomass concentration, pH, and carotenoids during cultivation

Throughout cultivation, 2 mL samples were collected for biomass and pH analysis. The samples were centrifuged at 1745×g for 10 min in a Cientec CT-5000 R centrifuge, Brazil, and the supernatant was reserved to measure pH according to AOAC [18] standards using a digital pH meter (Marte MB-10, Brazil). The sediment was washed with distilled water and centrifuged again in two additional steps. Biomass was quantified by absorbance reading at 620 nm and related to the standard curve constructed for yeast [20].

For carotenoid extraction, 0.05 g of dry biomass and 2 mL of dimethyl sulfoxide heated to 55 °C (cell wall rupture) were used, followed by vortex homogenization for 1 min every 15 min for 1h. After that, solid-liquid extraction was performed with 6 mL of acetone and vortex homogenization for another 1 min, and centrifuged at 1745× g for 10 min (Cientec CT-5000 R, Brazil). This process was repeated until the cell was bleached. In the liquid-liquid partition, 10 mL of 20% NaCl (m.v^− 1^) and 10 mL of petroleum ether were used, adding sodium sulfate to remove excess water, and it was left to stand until phase separation for recovery of the supernatant [20–22]. It was then quantified using spectrophotometry (Bioespectro SP-22, China) in the range of 448 nm for β-carotene and 474 nm for astaxanthin. The specific concentration used the specific absorptivity in petroleum ether for β-carotene of 2592 and astaxanthin of 2100 [11, 21, 23] calculated using Eq. 2 below, according to Davies [24]

2 $$\mathrm{T}\mathrm{C}=\frac{\mathrm{A}\mathrm{*}\mathrm{V}\mathrm{*}{10}^{6}}{{\mathrm{A}}_{\mathrm{C}\mathrm{M}}^{1\mathrm{\%}}\mathrm{*}100\mathrm{*}{W}_{\mathrm{S}\mathrm{a}\mathrm{m}\mathrm{p}\mathrm{l}\mathrm{e}}}$$

Where: TC is the specific concentration of total carotenoids (µg. g^− 1^), A is the absorbance, V is the volume (mL), w_sample_ is the dried cell weight (g), and ${\mathrm{A}}_{\mathrm{C}\mathrm{M}}^{1\mathrm{\%}}$ is the specific absorptivity.

### Recovery and drying of post-harvest biomass

The biomass was recovered through three washing cycles with distilled water at 3500 rpm, with durations of 20, 15, and 10 min in each cycle, respectively. The biomass was dried in a spouted bed, as adapted from [25], using a bed consisting of a cylindrical stainless steel column and a conical glass base (60°, 0.15 m height, 0.175 m diameter), operated in a stable spout regime at a velocity above the minimum spouting velocity (vjm) under the following conditions: biomass diluted to a moisture content below 95%, inlet air temperature of 95 °C, outlet air temperature of 65 °C, ΔT = 30 °C, compressed air at 101 kPa, and semi-continuous atomization of 0.2 kg·h⁻¹·kg⁻¹ of inert material. After drying, the biomass was ground to a particle size of 115 mesh [21].

### Centesimal composition of biomass

The dry biomass from the spout bed was characterized in terms of its centesimal composition following the AOAC [18] methodologies: proteins by the Kjeldahl method and ash by muffle furnace incineration. To determine lipids, the methodology described above was used, following the Bligh-Dyer method [26] adapted by Manirakiza, Covaci, and Schepens [27] with acid hydrolysis (2 M HCl) [28], and carbohydrates were calculated by difference.

### Lipid extraction by Bligh & Dyer

For lipid quantification, the methodology of Bligh & Dyer [26] adapted by Manirakiza; Covaci; Schepens [27], was tested. Two forms of extraction were performed: with chemical disruption of the cell wall using 5 mL of 2M hydrochloric acid (HCl) at 80 °C for 1h [28] and without acid hydrolysis. The samples with acid hydrolysis before the extraction step were cooled and centrifuged at 3560 x g for 15 min, discarding the supernatant. The extraction step was performed using methanol and chloroform in a ratio of 4:4 v.v^− 1^, with the chloroform being added in two portions, adding 3.6 mL of distilled water, and each addition followed by 2 min of vortexing. After this process, it was centrifuged at 3560x g for 15 min, recovering the oily fraction in a dry, pre-weighed flask. Re-extraction followed the same process as extraction, adapting only the use of solvent and volume, using only 4 ml of methanol: chloroform solution in a 1:9 (v.v^− 1^) ratio. The recovered lipid extract was evaporated until all solvent was completely removed. The results were expressed as % (w.w^− 1^) and determined according to Eq. 3 below [29].

3 $${\mathrm{Lipid}}\,{\mathrm{content}} = \frac{{{\mathrm{P}}_{{\mathrm{f}}} }}{{\mathrm{W}}}{\mathrm{x}}100$$

Where: P_f_ = mass of lipids extracted from the sample (g), W = sample weight (g).

### Determination of fermentation parameters

Fermentation parameters were determined with emphasis on the compositional characterization of the biomass at 144 h of cultivation. The specific growth rate (µ) was calculated according to Eq. 4 [30].

4 $$\mu \, = \,\frac{{(\ln {\mathrm{X}}_{2} - \ln {\mathrm{X}}_{1} )}}{{\left( {{\mathrm{t}}_{2} - {\mathrm{t}}_{1} } \right)}}$$

Where: X_2_ and X_1_ are the biomass concentration values measured in the culture; **t₂** corresponds to 0 h, and **t₁** corresponds to 24 h of cultivation.

Biomass, carotenoid, and lipid productivity were calculated according to Eq. (5) [31], (6), and (7) [14], respectively.

5 $$\mathrm{P}\mathrm{r}=\frac{{\mathrm{L}}_{\mathrm{f}}}{t}$$

6 $$\mathrm{P}\mathrm{r}\mathrm{B}=\frac{{B}_{\mathrm{f}}-{B}_{\mathrm{i}}}{t}$$

7 $$\mathrm{P}\mathrm{r}\mathrm{C}=\frac{{C}_{\mathrm{f}}-{C}_{\mathrm{i}}}{t}$$

Where: Pr corresponds to the overall lipid productivity (g. L⁻¹. h⁻¹). L_f_ represents the total lipid concentration (g. L⁻¹), PrB, corresponds to biomass productivity, ${B}_{f}$ e ${B}_{i}$ represent the final and initial biomass concentrations (g. L⁻¹) respectively. PrC corresponds to carotenoid productivity, ${C}_{f}$ e ${C}_{i}$ represent the final and initial carotenoid concentrations (g.L⁻¹), respectively, and t represents the total cultivation time (144 h).

### Characterization of microbial oil

#### Fatty acid profile

To obtain the methyl esters of the extracted lipids, the protocol described by Metcalfe et al. [32] was followed. The fatty acid profile was determined using a gas chromatograph equipped with a split/splitless injector and a Zebron ZB-FAME capillary column (20 m × 0.18 mm ID × 0.15 μm) and a flame ionization detector (FID) (Shimadzu, model 2010 Plus). N₂ was used as the carrier gas. The injector and detector temperatures were maintained at 260 °C and 250 °C, respectively. The chromatographic program lasted a total of 11 min, as follows: initial temperature of 80°C for 1.5 min, ramping to 160 °C at 40 °C·min⁻¹, then to 185 °C at 5 °C.min⁻¹, and finally to 260°C at 30°C·min⁻¹. Compounds were identified by comparing the retention times of the samples with those of fatty acid methyl ester standards (Supelco 37 components FAME MIX – Sigma-Aldrich), and quantification was performed based on the peak areas.

#### Nuclear magnetic resonance (NMR)

Nuclear magnetic resonance (NMR) spectroscopy was used to determine the iodine and saponification indices (Bruker High Field, model 400 MHz Ascend, Rheinstetten, Germany), equipped with a 7.05 T magnet (corresponding to 300 MHz for ¹H) and a 5 mm diameter probe. For analysis, 10% of lipids dissolved in 0.7 mL of CDCl₃ were transferred to the spectrometer. The standard ¹H-NMR sequence consisted of a 45° pulse, with an acquisition time of 3s, 1.3 relaxation delay,16 scans, and a spectral width of 14 ppm [33]. The data obtained from the integrated ¹H NMR spectra were processed using the PROTEUS program, as described by Reda and Carneiro [34].

#### Acidity and peroxide indices by titration

To determine the acidity index, titration was performed with 0.1N NaOH until a persistent pink color appeared for at least 30s [35]. Equation 8 determines the acidity in oleic acid (g.100g⁻¹).

8 $${\mathrm{Acidity}}\,{\mathrm{in}}\,{\mathrm{oleic}}\,{\mathrm{acid}}\left( {\frac{{\mathrm{g}}}{{100{\mathrm{g}}}}} \right) = \frac{{{\mathrm{V}}_{{{\mathrm{NaOH}}}} {\mathrm{*}}28.2{\mathrm{*N}}_{{{\mathrm{NaOH}}}} }}{{{\mathrm{Pa}}}}$$

Where: ${V}_{\mathrm{NaOH}}$is the volume used in titration, ${N}_{\mathrm{NaOH}}$is the normality of the NaOH solution, and ${P}_{a}$is the sample weight in grams.

The peroxide value [35] was determined by titration using an acetic acid–chloroform solution and KI (potassium iodide). The resulting solution was titrated with 0.1N sodium thiosulfate, added with 1% (m/v) starch indicator solution, and titration was continued until the blue color disappeared. The peroxide value was calculated using Eq. 9 and expressed in mEq O₂/kg of oil.

9 $$\mathrm{P}\mathrm{I}=\frac{\left({\mathrm{V}}_{\mathrm{a}}-{\mathrm{V}}_{\mathrm{b}}\right)\mathrm{*}\mathrm{N}\mathrm{*}1000}{\mathrm{P}\mathrm{a}}$$

Where ${V}_{a}$is the volume of thiosulfate used in titrating the sample, ${V}_{b}$is the volume of thiosulfate used in titrating the blank, $\mathrm{N}$ is the normality of the sodium thiosulfate solution, and Pa is the mass of the sample in grams.

#### Statistical analysis

The data obtained were processed using Statistica 7.0 software (StatSoft Inc., Tulsa, OK, USA). The results are presented as means ± standard deviation, when applicable. Statistical analyses included analysis of variance (ANOVA) to identify significant differences, followed by Tukey’s test at a 95% confidence level (*p* ≤ 0.05). For comparisons between two treatments, the t-test was applied with a significance level set at *p* < 0.05.

## Results and discussion

### Characterization of the substrate used in the production medium

The soybean molasses used in this study had an elemental composition of 30.63 ± 0.1% (w/w) carbon, 5.52 ± 0.1% (w/w) hydrogen, and 0.69 ± 0.0% (w/w) nitrogen. It should be noted that the nitrogen content was low compared to the other elements; however, this did not affect the growth of *P. rhodozyma*. A low nitrogen concentration leads to an increase in the carbon-to-nitrogen ratio (C/N), which was 44.4 in the production medium containing 100 g.L⁻¹ of soybean molasses. This ratio in culture media is essential for the enhancement or reduction of certain metabolites [36]. When the carbon content is high and the nitrogen content is low, as in the present study, lipid accumulation is favored [37].

Results similar to those obtained in this study were reported by Ferreira Filho, Burkert, and Burkert [14] in the characterization of soybean molasses, which contained 36.44% carbon, 7.39% hydrogen, and 0.75% nitrogen.

The centesimal composition presented in Table 1, the soybean molasses had a moisture content of 33.9% and show is rich in carbohydrates, consistent with the high sugar content that characterizes this byproduct. According to Gambarato et al. [38], these carbohydrates consist mainly of oligosaccharides such as raffinose and stachyose, which are advantageous for microbial cultivation, providing a rapid and readily available energy source. Among the other macromolecules, lipids were found in smaller amounts (2.6%). Similar values were reported by Oliveira, Michelon, and Burkert [15], who characterized soybean molasses for the cultivation of diazotrophic bacteria in exopolysaccharide production, reporting 3.45 ± 0.11% lipids, 7.51 ± 0.17% protein, 14.48 ± 0.20% ash, and 64.56% carbohydrates, totaling a dry extract of 69.61 ± 0.28%. Rakita et al. [39] reported lipid contents ranging from 4 to 20% in soybean molasses characterized for animal nutrition.

Regarding protein and ash, the values obtained were moderate for this substrate, highlighting the low nitrogen content of the medium and the presence of various minerals.

**Table 1:** Centesimal composition of soybean molasses

Component^a^	Component composition (% w.w^− 1^)
Lipids	2.6 ± 0.3
Proteins	7.3 ± 0.0
Ash	7.7 ± 0.0
Carbohydrates	82.5

^a^Dry weight base

Rakicka-Pustułka et al. [40] characterized soybean molasses for the cultivation of *Yarrowia lipolytica* as a supplement to a crude glycerol medium to enhance chiro-D-glucaric acid production, reporting variations in composition with 6.12% lipids, 6.84% proteins, 5.54% ash, and 33% carbohydrates, totaling a dry extract of 69.61 ± 0.28%. Therefore, variations in the centesimal composition of soybean molasses can occur depending on its source.

Its use as the sole substrate in the cultivation of *P. rhodozyma* was reported by Pacheco et al. [11], who demonstrated its potential for carotenoid production, thereby reinforcing its value as an agroindustrial substrate for the cultivation of this yeast.

### Cultivation using soybean molasses in a bench-scale bioreactor

**Fig. 1:**
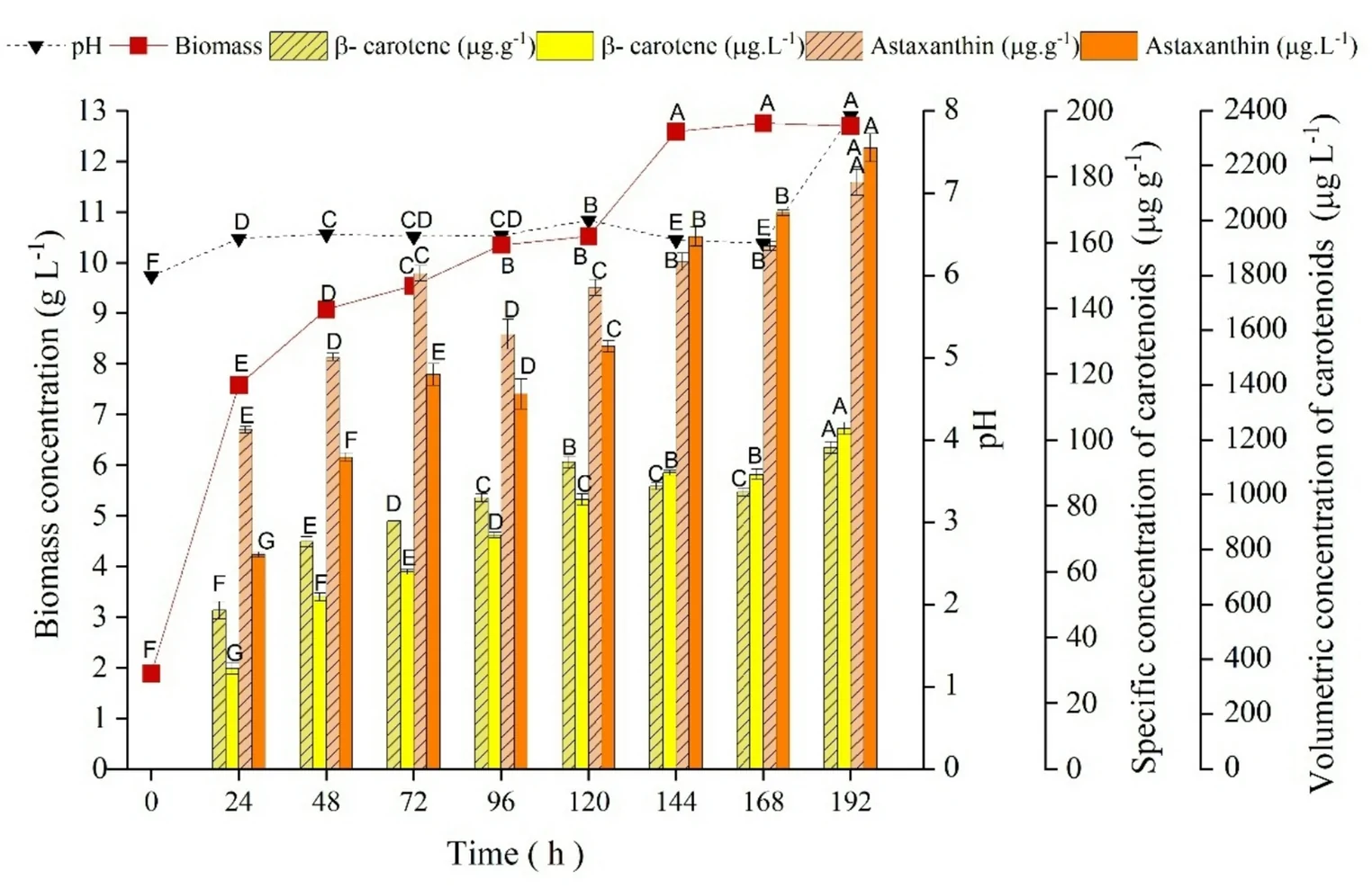
Growth kinetics of *P. rhodozyma* in a 5 L bench-scale bioreactor with 100 g.L⁻¹ soybean molasses, at 300 rpm, 1 vvm, 25 °C, and initial pH 6. Results are expressed as mean values ± standard deviations (*n* = 3); Different capital letters indicate significant differences (*p* < 0.05) throughout cultivation, after analysis of variance followed by Tukey’s test

The kinetics of *P. rhodozyma* cultivation in a bench-scale bioreactor using soybean molasses as the sole substrate are shown in Fig. 1. The highest biomass production occurred at 144h of cultivation, reaching a value of 12.59 ± 0.05 g.L⁻¹, with a carotenoid concentration expressed as β-carotene of 1081.67 ± 9.74 µg.L⁻¹ (85.91 ± 1.01 µg.g⁻¹) and astaxanthin of 1941.97 ± 34.81µg·L⁻¹ (154.23 ± 2.61 µg.g⁻¹). After 144 h of cultivation, no significant difference in biomass production was observed; however, the concentration of carotenoids remained stable (*p* < 0.05) between 144 and 168h, increasing at 172h. The specific and volumetric concentrations of β-carotene increased by 12.11% and 12.90%, respectively, and those of astaxanthin by 13.51% and 14.32%, respectively.

Silva et al. [8] used a bench-scale bioreactor with malt extract and yeast (YM) medium and applied magnetic fields to the culture under the same process conditions as in this study, achieving 8.74 g.L⁻¹ of biomass in 48 h of cultivation, with maximum production of β-carotene (4.67 mg.L⁻¹) and astaxanthin (4.51 mg.L⁻¹) in 96 h. Therefore, cultivation with soybean molasses enabled a 44% increase in biomass production; however, carotenoid production was about 70% lower than in this study. External stress from magnetic fields likely increased carotenoid production due to magnetoactivation in the phytoene synthase enzyme system, which promotes the formation of phytoene, a precursor of β-carotene and astaxanthin [8].

*P. rhodozyma* SFAS-TZ0 was cultured in sweet potato juice as the primary substrate and supplemented with 0.05% yeast extract, at 400 rpm and 1.4 vvm, with a working volume of 3L at 20°C [41], reaching 15.46 g·L⁻¹ of biomass and 6.04 mg·L⁻¹ of astaxanthin. These values were higher than the biomass and carotenoid production obtained in the present study, with differences of 27.66% and 32.10%, respectively, probably associated with the higher nitrogen content provided by medium supplementation for the synthesis of its compounds. This differs from the study that used soybean molasses as the sole carbon source, highlighting the potential of the by-product investigated to act as the primary and exclusive substrate in the cultivation of this yeast.

The concentration of carotenoids at 192 h increased due to accumulation during the stationary phase, reaching 97.75 ± 1.76 µg.g⁻¹ (1241.81 ± 22.32 µg.L⁻¹) for β- carotene and 178.32 ± 4.02 µg·g⁻¹ (2265.37 ± 51.04 µg.L⁻¹) for astaxanthin. These values can be explained by the fact that, when related to the stable biomass concentration after 144 h, it is evident that the yeast decreases cell growth and increases carotenoid synthesis due to the lack of nutrients (nitrogen content) in this cultivation phase, redirecting metabolism toward the synthesis of secondary metabolites such as carotenoids [42].

Pacheco et al. [11] studied the same yeast strain in a shaker-type system, observing similar behavior in YM medium cultivation, where the increase in secondary metabolites occurred at the end of cultivation (168 h), reaching 288.20 ± 6.01 µg.g⁻¹ (1420.60 ± 30.39 µg.L⁻¹) for astaxanthin and 162.64 ± 3.11 µg·g⁻¹ (801.70 ± 15.86 µg.L⁻¹) for β-carotene. It should be noted that cultivation in a bench bioreactor offers higher volumetric carotenoid production than in a shaker-type system, highlighting the importance of the bioreactor for optimizing and scaling up the fermentation process.

On the other hand, Urnau et al. [43] cultivated *P. rhodozyma* Y-17,268 using 100 g·L⁻¹ glycerol, 100 g·L⁻¹ corn maceration water, and 20 g.L⁻¹ parboiled rice water in a bioreactor with a working volume of 1L, under agitation at 250 rpm, aeration of 1.5 vvm, temperature of 25 °C, and initial pH of 4.0 for 96 h. Maximum total carotenoid production occurred at 60 h of cultivation, reaching 2.4 mg.L⁻¹ (639 µg.g⁻¹) and 2.45 g.L⁻¹ of biomass. After this period, carotenoid production decreased at 96 h to 1482.83 µg.L⁻¹ (489.25 µg.g⁻¹), with biomass of 4.88g·L⁻¹. The authors suggested that carotenoid degradation or the formation of secondary compounds (such as β-ionone) may have occurred.

The pH of the culture (Fig. 1) ranged from 6.00 ± 0.01 to 6.77 ± 0.06 up to 168 h, reaching a maximum value of 7.94 ± 0.04 at the final cultivation point. This behavior indicates that, during the stationary and cell death phases, amino acid deamination occurs, neutralizing organic acids accumulated during the growth phases. Additionally, nitrogen depletion at the end of cultivation and the variation in the C/N ratio favor ammonia production [44], corroborating the behavior observed in this study.

The volumetric oxygen transfer coefficient measures the efficiency with which oxygen from the air passes into the culture medium to become available to the cells. In the chosen agroindustrial medium and under the operating conditions, a k_L_a of 21.7 h⁻¹ was obtained, which is the value required for microbial biomass production in a laboratory bench bioreactor [45].

Studies with *Pichia guilliermondii* have demonstrated a positive correlation between k_L_a and biomass production, in which increased oxygen transfer enhanced dissolved oxygen levels, resulting in higher specific growth rates and dry cell mass concentrations. Under nitrogen-limited conditions, lipid accumulation increased, reaching 10.8 g.L⁻¹ of lipids at a k_L_a of 0.6 min⁻¹. This finding demonstrates how oxygen availability regulates respiratory metabolism and processes essential for biomass formation under aerobic conditions, while also favoring lipid biosynthesis [46].

A similar behavior was observed for *Rhodotorula glutinis*, which showed a dependence of the cell growth rate on kLa. Increased aeration improved oxygen transfer and significantly enhanced lipid productivity, reaching 25.40 g L⁻¹ at a k_L_a of 0.272 min⁻¹. However, it was also noted that high k_L_a values may redirect metabolism toward biomass formation rather than lipid accumulation, in addition to increasing foam formation, which may disrupt the fermentation process. For this reason, foam control and energy costs must be considered when defining aeration rates [47].

In the present study, although a k_L_a of 21.7 h⁻¹ (0.4 min⁻¹) was not directly optimized, the agitation and aeration conditions were sufficient to sustain yeast growth and the observed lipid production. Optimization of operational parameters, such as aeration and agitation rates, is necessary to improve oxygen transfer and maximize lipid production, while also considering energy consumption and foam control costs.

The use of 100 g.L⁻¹ soybean molasses in the cultivation of *P. rhodozyma* in the bench bioreactor provided significant results, reinforcing the hypothesis that this agroindustrial by-product is promising for microbial growth due to its composition. Based on the kinetic results, 144 h was defined as adequate for the end of cultivation, since during this period the maximum biomass concentration was reached, and the concentrations of carotenoids expressed as β-carotene and astaxanthin remained statistically equal to those obtained at 120 h and 168 h. Although maximum carotenoid production was reached at 192 h, interruption at the established period optimizes the balance between process duration and resource use.

In addition, soybean molasses showed no evidence of inhibitory effects when used as a substrate. According to Pacheco [11], when comparing the commercial medium, YM (Yeast–Malt), with soybean molasses for the cultivation of *P. rhodozyma*, higher biomass production was obtained using soybean molasses (7.21 g.L⁻¹) compared to the YM medium (6.25 g.L⁻¹). These results suggest that soybean molasses does not negatively affect the production of secondary metabolites, supporting its suitability as a substrate for the process. Other studies with diazotrophic bacteria using soybean molasses also showed no impairment in growth; on the contrary, it contributed to cell multiplication and reduced the need for culture medium supplementation, favoring the production of exopolysaccharides (EPS) [14]. These findings reinforce that soybean molasses is a potentially promising substrate for the cultivation of microorganisms aimed at obtaining secondary metabolites.

### Biomass composition

The biomass was characterized according to its centesimal composition (Table 2) to determine the metabolic orientation of the yeast under these production conditions and, in particular, to evaluate its biotechnological potential in lipid production. In *P. rhodozyma*, few studies have focused on biomass characterization, highlighting the importance of this study in assessing the potential of this microorganism. In the results presented, the lipid content (34.9%) was the second most abundant compound in the yeast, indicating that this microorganism has considerable potential for microbial oil production, as microorganisms that accumulate more than 20% lipid in their biomass are considered oleaginous [48].

When comparing the lipid content of the same *Phaffia rhodozyma* strain cultivated in complex media containing yeast extract, xylose, malt extract, and peptone under identical bioreactor conditions, Musaagy et al. [49] reported a total lipid yield of 23.72%, which is lower than that observed in the present study, highlighting the efficiency of soybean molasses as a substrate for microbial oil production.

Authors such as Rodrigues et al. [50] and Arrieira et al. [2], who studied *Rhodotorula mucilaginosa* cultivated in corn maceration water (3.4 g.L⁻¹) and sugarcane molasses (70 g.L⁻¹) at 25 °C, 180 rpm, and a C/N ratio of 143 in shaken flasks, reported lipid contents of 46.5% and 23.8%, respectively. In *P. rhodozyma*, few studies have reported centesimal composition. The study by Lai et al. [51] found 9.1% lipids in the biomass of *P. rhodozyma* Y119 cultivated on food waste (rice, fruits, vegetables, cooked food scraps, meat, fats, oils, and used lipids), a value lower than that obtained in the present study.

Other authors have reported the use of sugarcane molasses in pulsed fed-batch systems with C/N ratios ranging from 13.5 to 40, under controlled conditions of pH 5.8, 28 °C, 0.5 vvm, and 250 rpm, obtaining lipid contents of 41% for *Rhodosporidium toruloides*, 36% for *Rhodotorula glutinis*, 27% for *Rhodotorula minuta*, and 32% for *Lipomyces starkeyi* [31]. These results highlight the potential of sugarcane molasses for the cultivation of oil-producing yeasts associated with controlled feeding strategies. In contrast, the present study reveals the potential of soybean molasses without specific optimization of the culture medium, indicating that adjustments in operating conditions could significantly influence lipid accumulation by *P. rhodozyma*.

According to Niehus et al. [52], contextualizing the efficiency of lipid production by oleaginous yeasts requires a direct comparison with the yields of traditional vegetable oils. He observed that systems based on oil-producing yeasts can achieve yields up to 767 times higher than palm oil, which has a yield of 0.0018 .·m⁻².day⁻¹, and 36 times higher than microalgae (0.038 L.m⁻².day⁻¹), which face limitations related to photoperiod.

The study by Niehus et al. [52] with *Yarrowia lipolytica* reported oil production of 1.38 L.m⁻².day⁻¹, with a lipid content of 53% and a concentration of 3.8 g.L⁻¹. In comparison, the present study achieved 34.94% lipids in dry biomass (DCW) and a concentration of 4.23 g.L⁻¹. These results indicate that production with *Phaffia rhodozyma* is satisfactory and competitive with *Y. lipolytica*, and consequently superior to the yields obtained from palm oil and microalgae. In addition to corroborating this productive efficiency, the process offers a critical economic advantage by converting a low-value by-product into promising microbial oil, thereby eliminating the demand for new agricultural areas and avoiding direct competition with food production.

**Table 2:** Centesimal composition of *P. rhodozyma* cultured with 100 g.L^− 1^ of soybean molasses in a 5 L bench-scale bioreactor

Component^a^	Component composition(% w.w^− 1^)
	Dry biomass
Lipids	34.94 ± 0.3
Proteins	23.31 ± 0.4
Ash	4.79 ± 0.1
Carbohydrate	36.96

^a^Dry weight basis

The protein content in the yeast ranked third among the components of *P. rhodozyma*, indicating that although the fatty acid synthesis pathway was prioritized, cultivation with a C/N ratio of 44.4 yielded 23.3% protein, similar to the findings of Lai et al. [51], which underscores the potential of this yeast for producing this biomolecule.

*P. rhodozyma* has attracted attention in the literature due to its protein content, and authors such as Raita et al. [53] were able to increase the protein content of *P. rhodozyma* DSM 5626 to 31% of its biomass through inductive selection using herbicide, a value higher than that obtained in this study. Therefore, in this work, the biomass of *P. rhodozym*a contained a higher concentration of lipids than protein, but it can still be considered a relevant source of this macromolecule. These two biocomponents present in the yeast biomass directly reflect the influence of the culture medium. It should be noted that the techniques used by the aforementioned authors were optimized to produce a specific biocomposite. However, in the present study, the agroindustrial medium was used as the sole substrate, and the culture medium was not optimized, highlighting the potential of soybean molasses for lipid and protein production.

Regarding ash content, which represents the mineral fraction of the biomass, a value of 4.8% was obtained, indicating the presence of inorganic compounds from the yeast and/or the substrate used in the production medium (Table 1). In studies with *Yarrowia lipolytica* strains ATCC 9773 and NRRL Y-50,997, Torres-Añorve and Sandoval [54] reported ash contents of 5.80% and 6.88%, respectively, higher than those found in *P. rhodozyma*, which may indicate a lower level of inorganic impurities in this microorganism [55]. Carbohydrates were the most abundant compound in the yeast, representing polysaccharides present in the cell wall as well as some reserve compounds [56]. Finally, the biomass had a moisture content of 3.9%, indicating high efficiency in drying and recovery of the solid fraction.

Thus, it can be stated that *P. rhodozyma* cultivated in soybean molasses exhibits a high biological value due to the proportions of biocomponents it provides, further emphasizing the potential of soybean molasses as a substrate for this yeast and for future applications in other oil-producing yeasts.

### Influence of Bligh and Dyer extraction with and without acid hydrolysis

**Fig. 2:**
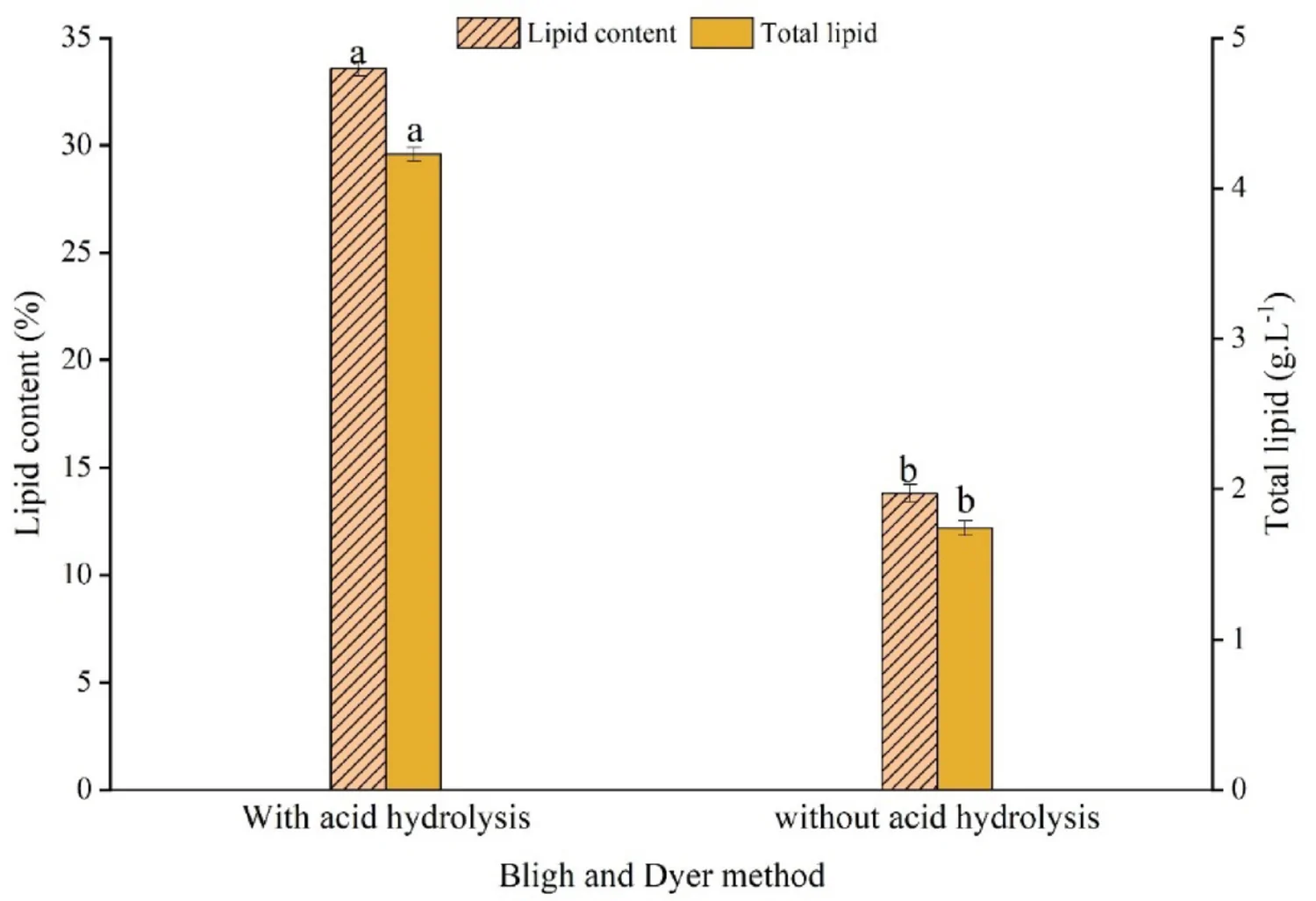
Lipid recovery by the Bligh and Dryer method with and without acid hydrolysis of *P. rhodozyma* in a 5 L bench-scale bioreactor with 100 g/L soybean molasses at 300 rpm, 1 vvm, 25 °C, and initial pH 6. Results are expressed as mean values ± standard deviations (*N* = 3); Different lowercase letters indicate significant differences (*p* < 0.05) between the two extraction methods by Bligh and Dryer according to the t–test

In the recovery of lipids produced by this yeast after spouted drying, two extraction approaches were evaluated: the conventional Bligh and Dyer method without acid hydrolysis, and a method incorporating acid hydrolysis prior to extraction. This strategy had been previously applied to this strain of *P. rhodozyma* to recover intracellular biocomponents using chemical and physical methods for cell disruption [28]. The Bligh and Dyer method without acid hydrolysis in this study allowed the evaluation of whether the spouted drying process, which applies constant mechanical stress, would be sufficient to disrupt the cell wall. The results presented in Fig. 2 show that lipid recovery with acid hydrolysis was 33.58 ± 0.34% (4.23 ± 0.04 g.L⁻¹), whereas recovery without acid hydrolysis was 13.80 ± 0.40% (1.74 ± 0.05 g.L⁻¹), confirming that acid hydrolysis increased cell wall disruption efficiency by 58.90%.

Some authors have explored alternative mechanical lysis methods, such as conventional sonication, achieving 99.8% lipid recovery, as well as hydrothermal treatment at 120°C for 60 min, resulting in 84.6% lipid recovery for *Rhodotorula toruloides* Y-6987 [57].

These results differ from those of the present study, in which mechanical lysis performed by the jet bed was insufficient to recover the lipid content of the biomass. Therefore, efficient alternatives are required to minimize the use of highly toxic chemical reagents in cell disruption and extraction, which are essential for the recovery of microbial oils for future food applications.

With regard to costs, authors such as Caporusso et al. [58] indicate that acid pretreatments have minimal expenses and that hexane, often used for oil extraction, contributes negligibly due to its high recycling rate, close to 100%. Bonatsos et al. [59] points out that industrial-scale production of single-cell oils (SCO) faces significant limitations in terms of operating costs, raw materials, and utilities. The author reports that 70% of total expenses arise from fixed capital, which includes bioreactors, agitators, and aeration systems. The second-largest expense is raw materials, with glucose and yeast extract for cultivation accounting for approximately $23.76 million per year. Utilities costs amount to around $3.97 million per year, presenting challenges due to substantial fixed capital investment and high operating costs, particularly for raw materials and utilities.

According to Renegar et al. [60], the use of waste streams can significantly reduce costs by up to $15 million per year. Thus, the present study offers a cost advantage regarding raw materials, as soybean molasses is employed as the only substrate for cultivation, eliminating the need for glucose and yeast extract. However, fixed capital costs remain a challenge in microbial lipid production.

### Fermentation parameters of microbial oil

A biomass concentration of 12.59 g.L⁻¹ was obtained, with a productivity of 0.06 g.L⁻¹.h⁻¹. At this biomass concentration and productivity, a substantial lipid accumulation capacity was observed, reaching 34.9% lipids per gram of dry biomass, corresponding to a lipid productivity of 0.03 g.L⁻¹.h⁻¹ (Table 3).

**Table 3:** Fermentation parameters for the Specific growth rate (h^− 1^), productivity of biomass (g. L⁻¹.h⁻¹), carotenoids (µg. L^− 1^.h^− 1^) and lipids (g.L⁻¹.h⁻¹) by *P. rhodozyma*

Parameters	Obtained value
Specific growth rate (h^− *1*^)	0.06
Biomass productivity (g.L^− 1^.h^− 1^)	0.07
Volumetric carotenoid productivity (µg. L^− 1^.h^− 1^)	Astaxanthin: 13.49
	β-carotene: 7.51
Lipid productivity (g.L^− 1^.h^− 1^)	0.03

The productivity values were similar to those reported by Urnau et al. [41], who used agro-industrial substrates for cultivation and achieved results comparable to those obtained in the present study, with a maximum biomass productivity of 0.05 g.L⁻¹.h⁻¹ and a specific growth rate of 0.39 h⁻¹. However, astaxanthin productivity was higher in the cited study, reaching 28 µg.L⁻¹.h⁻¹. It should be considered that the authors employed a fed-batch system and multiple substrates to supplement carbon and nitrogen sources, which contributed to increased productivity. In the present study, although lower values were obtained for some parameters, it is noteworthy that only a single substrate was used under batch cultivation conditions, while still achieving a slightly higher biomass productivity than that reported by Urnau et al. [41].

Regarding lipid productivity, the values obtained in the present study are consistent with those typically reported for simple batch cultivations (Table 4). Vieira et al. [31], who evaluated lipid production by *Rhodosporidium toruloides* CCT 0783, *Rhodotorula glutinis* CCT 2182, *Lipomyces starkeyi* DSM 70,296, and *Rhodotorula minuta* CCT 1751 cultivated using Brazilian sugarcane molasses under pulsed fed-batch conditions with salt supplementation to achieve the desired carbon-to-nitrogen ratio, reported lipid contents of 44%, 36%, 32%, and 27%, respectively. The lipid productivities achieved were 0.41, 0.27, 0.13, and 0.11 g.L⁻¹.h⁻¹, respectively. Therefore, the lipid contents obtained in the present study are within the range reported in the cited work, although lipid productivity was lower.

Kuttiraja et al. [52], who studied *Yarrowia lipolytica* SKY7 cultivated in glycerol for 72 h at a C/N ratio of 75 in a 15 L bioreactor, achieved a lipid yield of 54.1% and a specific growth rate of 0.15 h⁻¹, decreasing to 0.04 h⁻¹ after 72 h, values higher than those observed in the present study. Nevertheless, despite the lower growth rate obtained here, a lipid yield of 34.94% was achieved at a C/N ratio of 44.4.

Therefore, it is suggested that the high lipid content observed in the present study may be associated with metabolic redirection toward lipid accumulation, particularly under possible nutrient limitation, a behavior similar to that described for *Yarrowia lipolytica* SKY7 [61].

The results demonstrate the promising performance of *P. rhodozyma* as a microbial oil producer and indicate the potential for maximizing lipid production by *P. rhodozyma*. In this context, it is emphasized that the present study establishes an initial foundation for understanding its lipid metabolism in this alternative substrate.

Table 4 below presents a comparison between the present study and literature studies regarding lipid production under different cultivation conditions.

**Table 4:** Lipid production by oleaginous yeasts considering strain, substrate, cultivation system, lipid content (%), and lipid productivity (g. L⁻¹.h⁻¹)

Yeasts	Substrate	Cultivation conditions	Lipid (DW %)	Lipid productivity (g.L⁻¹.h⁻¹)	References
*P. rhodozyma* NRRL Y-17,268	Soybean molasses	300 rpm, 1vvm 25 °C, pH 6	34.9	0.03	This study
*R. mucilaginosa* CCT 7668	Sugar cane molasses, corn steep liquor	180 rpm, 25 °C, pH 6	24.5	–	Arrieira et al. [2]
*Yarrowia lipolytica* NRRL Y-1095	Glucose, yeast extract, KH₂PO₄,MgSO₄0.7 H₂O,	180 rpm, 28 °C, pH 6	17.13	–	Rasia et al. [62]
*Rhodosporidium babjevae* DBVPG 8058	Corn stover, corn stover hydrolysate, (NH_4_)_2_SO_4_, KH_2_PO_4_, MgSO_4_	120 rpm, pH 5.5, 25 °C	18.5	0.04	Fontes et al. [63]
*Trichosporon oleaginosus DSM 11,815*	Corn stover, corn stover hydrolysate, (NH_4_)_2_SO_4_, KH_2_PO_4_, MgSO_4_	120 rpm, pH 5.5, 25 °C	24.6	0.14	Fontes et al. [63]
*Trichosporon oleaginosus DSM 11,815*	Corn stover, corn stover hydrolysate, (NH_4_)_2_SO_4_, KH_2_PO_4_, MgSO_4_	30°C, pH 5.5, 1 vvm, stirring rate was set manually	42.3	0.06	Fontes et al. [63]
*Rhodosporidium paludigenum* NCYC *2663*	Sucrose, yeast extract, (NH₄) ₂SO_₄_ KH₂PO₄,Na₂HPO₄, MgSO₄ · 7 H₂O, CaCl₂·2 H₂O, FeCl₃·6 H₂O_,_ MnSO₄ · H₂O (0,06 _) e_ ZnSO₄ _·_ 7 H₂O	200– 400 rpm, air flow rate of 1.5– 2.5 L. min^− 1^, pH 6	36.1	–	Sereti et al. [64]

Thus, the results obtained are promising for lipid production by oleaginous yeasts. Although lipid productivity presented lower values, they are consistent with those reported in the literature. Another relevant aspect concerns the substrate used, since the lipid content achieved in this study can be considered satisfactory when compared to studies that employed medium supplementation to adjust the C/N ratio and applied additional process optimization strategies. Therefore, a significant lipid accumulation was observed, highlighting the lipogenic metabolic potential of *P. rhodozyma* cultivated using soybean molasses as the sole substrate.

### Characterization of oil extracted from *P. rhodozyma*

#### Fatty acid profile

The identification of fatty acids in microbial oil (Table 5) is essential to understand its technological and nutritional properties. It can be observed that monounsaturated fatty acids are predominant, representing 68.29%, among which oleic acid is the most abundant, accounting for 66.98% of the total monounsaturated content. Gadoleic acid was present at lower concentrations (0.46%). Regarding saturated fatty acids, which represent 19.44% of the total oil, palmitic acid was the most abundant at 15.21%, while myristic acid was present at a lower concentration of 0.43%. Among polyunsaturated fatty acids, which totaled 12.27% of the oil, linoleic acid was predominant at 10.83%, whereas linolenic acid was present at a lower concentration of 1.44%. Thus, the saturated/unsaturated ratio is 0.24, indicating that the microbial oil under study is rich in unsaturated fatty acids.

Feng et al. [65] determined the fatty acid profile of *P. rhodozyma* D3 and identified 12 saturated fatty acids, 5 monounsaturated fatty acids, and 7 polyunsaturated fatty acids, with oleic acid at 26.26 mg.g⁻¹ and linoleic acid at 4.04 mg.g⁻¹, both lower than the values found in the strain under study. Among the saturated fatty acids reported by the authors, palmitic acid was the most abundant at 5.36 mg.g⁻¹ and stearic acid at 0.952 mg.g⁻¹, also below the quantities quantified for this strain.

Zhang et al. [66] applied TiO₂ as a stress factor in *P. rhodozyma* PR106 to enhance astaxanthin production and subsequently investigated its relationship with fatty acid synthesis. They observed that increased astaxanthin levels reduced the amounts of saturated fatty acids, such as palmitic and stearic acids, and increased the production of unsaturated fatty acids, including oleate, linoleate, and α-linolenate, with the highest increase observed for linoleate.

**Table 5:** Fatty acid profile of oil from *P. rhodozyma* Biomass Cultivated in a 5 L Bioreactor with 100 g.L⁻¹ Soybean Molasses, at 300 rpm, 1 vvm, 25 °C, and Initial pH 6

Fatty acid profile% (m. m^− 1^)		
Fatty acids	Type	Percentage composition
Myristic acid (C14:0)	SFA	0.43 ± 0.00
Palmitic acid (C16:0)	SFA	15.21 ± 0.10
Palmitoleic acid (C16:1)	MUFA	0.85 ± 0.01
Stearic acid (C18:0)	SFA	2.83 ± 0.26
Oleic acid (C18:1 C)	MUFA	66.98 ± 0.46
Linoleic acid (C18:2 C)	PUFA	10.83 ± 0.27
Linolenic acid (C18:3 C)	PUFA	1.44 ± 0.09
Gadolic acid (C20:1)	MUFA	0.46 ± 0.02
Lignoceric acid (C24:0)	SFA	0.97 ± 0.07

*SFA* Saturated, *MUFA* Monounsaturated, *PUFA* Polyunsaturated

The predominance of unsaturated fatty acids in the oil produced by *Phaffia rhodozyma* strain NRRL Y-17,268 cultivated on soybean molasses underscores the underexplored lipid-producing potential of this species. Traditionally, this yeast has been primarily associated with carotenoid biosynthesis [9, 11, 17], has been little studied in relation to its capacity for lipid accumulation using agro-industrial by-products, which induce fatty acid synthesis through *de novo* lipogenesis [67]. The results indicate the technological and nutritional relevance of the oil, which, with studies monitoring dissolved oxygen in cultivation (DO), will enable future applications.

#### Nuclear magnetic resonance (NMR)

**Fig. 3:**
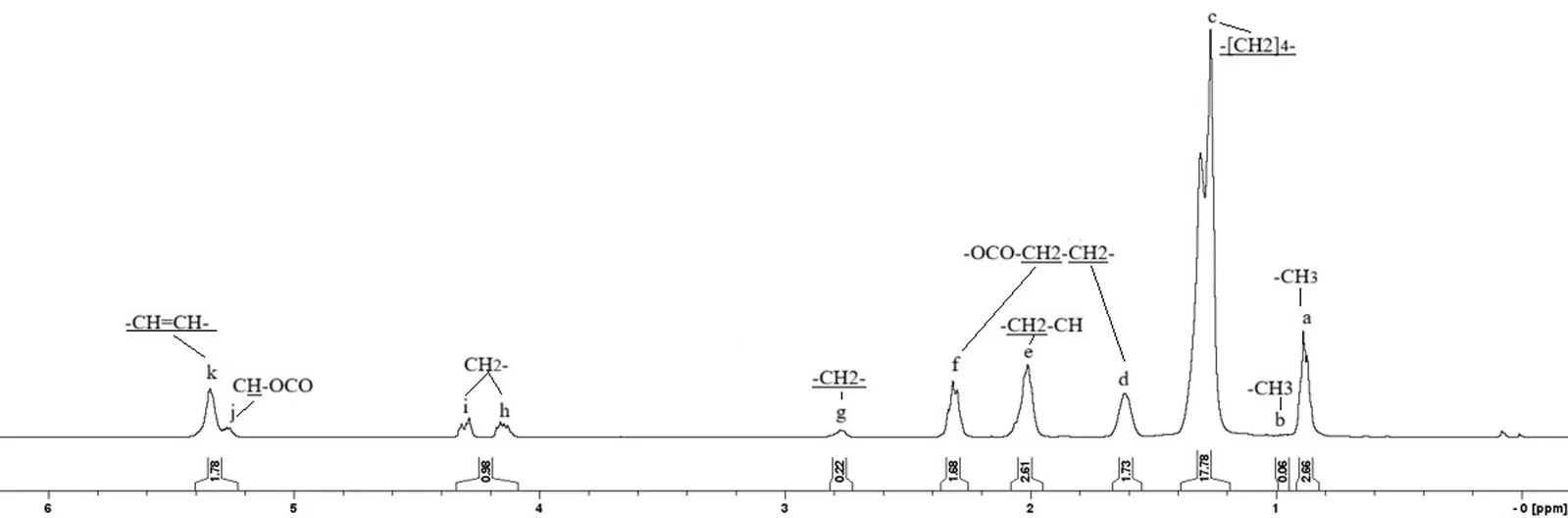
Nuclear Magnetic Resonance of *P. rhodozyma* oil cultivated in a 5 L bench-scale bioreactor with 100 g.L^− 1^ of soybean molasses

NMR serves as a tool for identifying functional groups in triglycerides. Furthermore, this method enables the determination of chemical parameters such as the acidity index, ROA ratio (oleic/linoleic acid ratio), iodine value, and saponification number. The microbial oil from *P. rhodozyma* was characterized by NMR (Fig. 3). In this analysis, the chemical shifts and peak integrations were correlated with fatty acid profile values obtained by chromatography. The identification of NMR spectrum signals followed the criteria proposed by Reda and Carneiro [34]. The most intense peak (c) in the range of 1.15–1.40 ppm corresponds to the methylene protons of triacylglycerol fatty acids, with a relative area of 17.78. This signal is associated with the predominance of long aliphatic chains, mainly reflecting the high proportion of oleic acid (66.98%), which was the principal fatty acid identified in the oil.

Peak d indicates β-carboxylic protons in the 1.50–1.70 ppm range. Another notable peak is f, observed in the 2.22–2.34 ppm range, corresponding to α-carboxylic protons adjacent to the carboxyl group. Peak g, in the range of 2.7–2.8 ppm, represents internal allylic protons, which are typical of polyunsaturated fatty acids such as linolenic acid (1.44%) and linoleic acid (10.83%). Peak K corresponds to olefinic protons with chemical shifts between 5.26 and 5.40 ppm, indicating the presence of double bonds in the acyl chains of fatty acids. Peak j represents the methylene H-2 protons of glycerol, characteristic of the central carbon in glycerol of triacylglycerides, confirming that the oil under study contains intact triacylglycerols. The integration of peaks a + b (methyl protons) reflects the saturated fraction of the molecule, which may be associated with myristic acid (0.43%), palmitic acid (15.21%), and lignoceric acid (0.97%).

The integrated peaks, correlated with the lipid profile, indicated an acidity index of 5.02 mg KOH.g⁻¹ and 2.52% free fatty acids, revealing a high content of these compounds. The maximum allowed acidity for cold-pressed and unrefined vegetable oils is 4 mg KOH.g⁻¹ [68], and the observed value exceeded this limit. However, filtration using polyvinylidene fluoride hollow fiber membranes [69] or enzymatic deacidification [70] can reduce free fatty acids in the oil, ensuring compliance with regulatory standards.

The iodine value was determined as 74.00 g I₂.100 g⁻¹ of oil, consistent with the presence of unsaturated fatty acids such as oleic and linoleic acids, indicating a medium to high degree of unsaturation. The calculated saponification number of 161 mg KOH.g⁻¹ confirms the triacylglyceride structure of the oil and indicates the presence of C16 and C18 chain fatty acids. Arrieira et al. [2] characterized microbial oil from *R. mucilaginosa*, reporting an iodine value and saponification number of 79.8 g I₂.100 g⁻¹ and 178.2 mg KOH.g⁻¹, respectively. These values are lower than those found in this study, likely due to differences in yeast strain and culture medium.

The ROA value evaluates the degree of unsaturation and oxidative stability of the oil. Using the NMR method, it was 2.30, indicating that oleic acid is 2.3 times more abundant than linoleic acid. This higher proportion of monounsaturated fatty acids suggests greater oxidative stability, as these fats are less susceptible to lipid auto-oxidation [71].

#### Acidity and peroxide

The acidity and peroxide indices are essential parameters for assessing the quality of oil for consumption (Table 6). The acidity content was re-evaluated because NMR analysis can overlap or obscure free fatty acids, particularly when some are bound to glycerol or other compounds. The peroxide value was 3.19 meq O₂.kg⁻¹ of oil and 3.60 meq O₂.kg⁻¹ of oil for biomass stored for 2 days and 2 months, respectively. Therefore, the peroxide value is within acceptable limits, considering 10 meq O₂.kg⁻¹ as a reference for refined vegetable oils [69]. This indicates that the oil exhibits low oxidative degradation and that there is no significant difference after 2 months of biomass storage. Bencresciuto et al. [72] evaluated the peroxide value of *Lipomyces starkeyi* oil fermented with dairy effluents at two temperatures (25 and 30°C), reporting peroxide values of 0.14 meq O₂.kg⁻¹ at 30°C and none at 25°C. These low peroxide values are comparable to those observed in this study, demonstrating that microbial oils have minimal lipid oxidation and may be promising candidates for food applications.

Regarding the acidity parameters, it can be observed that biomass stored for 2 months exhibited a 50% increase in the acidity content of the extracted oil. Moreover, the acidity in biomass stored for 2 days (7.00 ± 0.01 g.100 g⁻¹ oleic acid) was 63.86% higher than that determined by NMR, indicating a high content of free fatty acids. This difference between the NMR and titrimetric analyses is likely due to the larger amount of biomass required for the titrimetric method, which involves longer oil storage compared to NMR. Variations in acidity in the stored biomass may be explained by the continued activity of lipases after drying; Thus, as the lipase/water/lipid interface decreased, it promoted the action of enzymes that, under suitable conditions, these enzymes hydrolyze triacylglycerides present in the biomass of carotenogenic yeasts [73], resulting in an increase in free fatty acids.

Even though the oil is extracted rapidly, the acidity content is high at the beginning of the process. It can therefore be assumed that extraction methods involving chemical or enzymatic hydrolysis may increase the free fatty acid content [74], since the breakdown of the cell wall can also result in the hydrolysis of triacylglycerides, thereby increasing acidity.

**Table 6:** Acidity and peroxide Indices of *P. rhodozyma* oil cultivated in a 5 L bench-scale bioreactor with 100 g·L⁻¹ soybean molasses

Storage time for dry biomass	Peroxide (mEq O2. Kg^− 1^ of oil)	Acidity in oleic acid (g. 100 g^− 1^ oleic acid)
2 days	3.19 ± 0.18^a^	7.00 ± 0.00^a^
2 months	3.60 ± 0.40^a^	14.00 ± 0.01^b^

Different lowercase letters indicate significant differences (*p* < 0.05) between the two biomasses with different storage by the t-test.

Thus, it can be stated that in both cases, the high acidity observed in the oil is associated with the partial hydrolysis of triacylglycerols, which was likely favored by the activity of lipases present in the biomass. These enzymes are capable of hydrolyzing triacylglycerols in aqueous media, releasing free fatty acids, monoacylglycerols, diacylglycerols, and glycerol. Furthermore, lipases exhibit structural stability and maintain activity across different pH ranges, do not require cofactors, and, most importantly, their activity may persist in the absence of prior biomass inactivation before extraction [75].

Therefore, the presence of residual moisture during storage may have promoted enzymatic hydrolysis, contributing to the increased acidity. Additionally, certain extraction conditions may have enhanced interactions at the hydrophobic/hydrophilic interface, thereby intensifying lipase activity and increasing the release of free fatty acids [76]. Collectively, these factors suggest that biomass storage and processing conditions during oil recovery may explain the acidity values observed in this study.

Burgstaller et al. [77] reported an acidity index in single-cell oils from *Apiotrichum brassicae* and *Pichia kudriavzevii* based on acetic and propionic acids of 4.3 mg KOH·g⁻¹, a high value similar to that observed in the present study, which does not comply with the reference parameter. Currently, there are no official acidity or peroxide parameters for microbial oils due to their classification as novel foods and novel food ingredients, which necessitates a flexible and comprehensive safety assessment, given the uniqueness of the source organisms [78]. However, according to Brasil [67], microbial oils should meet the limits established for vegetable oils intended for consumption. Given the high acidity levels of the oil, it is essential to explore less aggressive extraction techniques to mitigate this issue, as well as storage methods that prevent lipase activity. Another approach to reduce acidity is refining; however, priority should be given to processes that do not compromise the sensory and nutritional properties of the oil.

## Conclusion

The results of this study demonstrated that soybean molasses is an efficient and low-cost substrate for the cultivation of *P. rhodozyma*. This by-product enabled the production of microbial biomass (12.59 g.L⁻¹) rich in carotenoids, such as astaxanthin and β-carotene, as well as lipids (33.8%) and proteins (23.3%), consolidating its potential as a producer of multiple value-added bioproducts. In addition, the high C/N ratio (44.39) of the culture medium favored lipid accumulation, positioning *P. rhodozyma* as an oil-producing yeast with a lipid recovery of 4.2 g·L⁻¹. The oil produced by the yeast was predominantly composed of monounsaturated fatty acids, accounting for 68.29%, mainly oleic acid (66.98%), along with 12.27% polyunsaturated fatty acids, which contributed to greater oxidative stability and a high degree of unsaturation.

Although the biomass remained stable during storage and acid hydrolysis was required to maximize lipid recovery, improving the acidity index remains a technical challenge to meet regulatory standards for food applications. Therefore, the use of this by-product as a substrate for *P. rhodozyma* cultivation represents a promising approach to reducing production costs and environmental impacts associated with microbial oil production, providing a solid basis for future scale-up studies for food applications and positioning *P. rhodozyma* as a natural, biotechnological, sustainable, and functional source.

## Supplementary Information

Below is the link to the electronic supplementary material.

Supplementary Material 1

## Data Availability

No datasets were generated or analysed during the current study.
