# Supplementary material for: Soybean molasses: a promising substrate for the production of microbial oil by Phaffia rhodozyma
Source: Bioprocess Biosyst Eng. 2026 Apr 15;49(9):2471–86. doi: 10.1007/s00449-026-03312-y (PMC13582171; doi:10.1007/s00449-026-03312-y)
Supplement: Supplementary file 1 [file 449_2026_3312_MOESM1_ESM.docx]

**Supplementary Material**

**Soybean molasses: a promising substrate for the production of microbial oil by *Phaffia rhodozyma***

^1^Shirley Denisse Ccori Ponce* <https://orcid.org/0009-0004-8896-3369>

^2^Luiz Henrique Han [https://orcid.org/](https://orcid.org/0009-0004-8896-3369)0000-0001-5993-9336

^2^Luiz Antonio de Almeida Pinto [https://orcid.org/](https://orcid.org/0009-0004-8896-3369)0000-0002-4477-0686

^3^Carlos André Veiga Burkert <https://orcid.org/0000-0003-0169-0894>

^1^Janaina Fernandes de Medeiros Burkert <https://orcid.org/0000-0002-8504-5376>

^1^ Sensory Analysis and Quality Control Laboratory, School of Chemistry and Food, Federal University of Rio Grande, Rio Grande, 96203-900, Brazil.

^2^ Drying Laboratory and Industrial Technology Laboratory, School of Chemistry and Food, Federal University of Rio Grande, Rio Grande, 96203-900, Brazil.

^3^Bioprocess Engineering Laboratory, School of Chemistry and Food, Federal University of Rio Grande, Rio Grande, 96203-900, Brazil.

* Corresponding author: [ccoriponceshirleydenisse@gmail.com](mailto:ccoriponceshirleydenisse@gmail.com)

**Number of Pages: 2**

**Number of Figures: 1**

**Figure 1S** – Biomass standard curve used for *Phaffia rhodozyma* NRRL Y-17268.
